# “Oh my word, so overwhelmed”: Exploring the patient and family experience with diagnosis and treatment decision-making in pediatric CNS tumors

**DOI:** 10.1093/noajnl/vdaf202

**Published:** 2025-09-16

**Authors:** Gabrielle Helton, Will Daley, Gianni Solis, Claire Miller, Daniel Pacheco, Adam L Green

**Affiliations:** Morgan Adams Foundation Pediatric Brain Tumor Research ­Program, University of Colorado School of Medicine, Aurora, ­Colorado; Morgan Adams Foundation Pediatric Brain Tumor Research ­Program, University of Colorado School of Medicine, Aurora, ­Colorado; Morgan Adams Foundation Pediatric Brain Tumor Research ­Program, University of Colorado School of Medicine, Aurora, ­Colorado; Morgan Adams Foundation Pediatric Brain Tumor Research ­Program, University of Colorado School of Medicine, Aurora, ­Colorado; University of Colorado Cancer Center, Aurora, Colorado; Morgan Adams Foundation Pediatric Brain Tumor Research ­Program, University of Colorado School of Medicine, Aurora, ­Colorado; Neuro-Oncology Program, Children’s Hospital Colorado, Aurora, Colorado; University of Colorado Cancer Center, Aurora, Colorado

**Keywords:** experience of diagnosis | healthcare communication | healthcare disparities | pediatric brain tumors | treatment decision-making

## Abstract

**Background:**

Pediatric central nervous system (CNS) tumors are the leading cause of cancer-related mortality in children, with survival outcomes significantly influenced by racial, ethnic, and socioeconomic disparities. These disparities may arise from delayed diagnosis, unequal access to care, and challenges in navigating complex treatment decisions, including clinical trial enrollment. This study explores the experiences of a diverse cohort of families from symptom onset through treatment initiation, focusing on their perspectives on diagnosis, treatment discussions, and decision-making processes.

**Methods:**

This qualitative study involved semi-structured interviews with families of children diagnosed with pediatric CNS tumors. Transcripts were analyzed by two separate coders using thematic analysis.

**Results:**

Four major themes were identified: Experience of diagnosis, Treatment discussion, Treatment decision, and Communication. Families described the emotional toll of diagnosis, marked by uncertainty, shock, and urgency. Many reported difficulties understanding complex medical information and accessing advanced treatment options. Treatment decisions were influenced by perceptions of therapeutic efficacy, anticipated side effects, and available family-level resources. Variations in perceived barriers to care highlighted disparities in support-seeking behaviors, emphasizing the need for personalized communication and tailored resources.

**Conclusion:**

This study provides a nuanced understanding of pediatric CNS tumor care by centering patient and family experiences. Findings underscore the need for targeted interventions to improve access to innovative treatments, support informed decision-making, and enhance communication. Future research should incorporate quantitative methods to validate findings and develop scalable solutions that address structural and informational barriers, ultimately reducing disparities and improving family experiences in pediatric CNS tumor care.

Key Points

- Families experience logistical challenges navigating pediatric CNS tumor care.
- Clear, tailored communication improves decision-making and treatment preparedness.
- Families’ treatment decisions vary based on unique priorities and circumstances.

Importance of the Study

This study highlights the critical role of communication and support in navigating pediatric CNS tumor care. Families experience significant emotional and logistical challenges from diagnosis through treatment, often struggling with complex medical information and access to advanced therapies. Disparities in care may arise from variable diagnostic experiences, differing levels of health literacy, and inconsistent communication, impacting decision-making and clinical trial enrollment. By centering family perspectives, this study underscores the need for tailored, proactive communication strategies and consistent team members doing that communication to improve trust, access, and informed decision-making. Findings emphasize the importance of addressing structural and informational barriers to reduce disparities and enhance the family experience. Future research should integrate quantitative methods to validate these findings and develop scalable interventions that ensure equitable, family-centered care for children with CNS tumors.

Central nervous system (CNS) cancers recently surpassed leukemia as the leading cause of cancer-related death among children, with racial, ethnic, and socioeconomic disparities contributing to survival outcomes in this population.^1^ Black and Hispanic children with CNS cancers face significantly higher mortality risk compared to non-Hispanic White children, and children from high-poverty areas also experience poorer survival outcomes.^2^^,^^3^ While these disparities are well-documented at the population level, limited research explores the lived experiences of patients and families, particularly regarding how these factors shape access to treatment. Understanding these family-level experiences is critical for identifying actionable steps to mitigate these disparities and improve equity in pediatric oncology care.

In pediatric oncology, timely diagnosis and access to high-quality treatments are essential for improving outcomes across cancer types.^4^^,^^5^ However, children from underserved populations often face significant barriers to receiving optimal care. Research into adult CNS cancers shows that patients with private insurance fare better in treatment outcomes, highlighting how socioeconomic factors influence access to quality care and survival.^6^ While some studies address these disparities in adult cancers, similar work in pediatric CNS tumors remains scarce. Our work builds on the existing literature by specifically focusing on post-diagnosis factors that may contribute to survival disparities. This study is among the first to explore experiences at the family and individual levels, offering insights that could inform targeted interventions to equalize access to cutting-edge treatments and improve survival outcomes across diverse populations. Furthermore, our study aims to investigate the factors influencing treatment decisions for newly diagnosed or recurrent CNS tumors and evaluate the barriers families face. Our study delves into the experiences of patients and families from the onset of symptoms through the initiation of treatment, providing in-depth insights into the diagnostic journey and treatment discussions. By conducting qualitative interviews with a small, yet diverse cohort of families, we seek to identify modifiable barriers in treatment planning and trial participation, which could be addressed through hospital, community, and cancer center resources. This research also aims to lay the groundwork for future quantitative studies assessing the impact of such interventions, with the goal of identifying modifiable barriers to help mitigate survival disparities among the pediatric CNS tumor population.

## Materials and Methods

### Study Design and Population

This prospective, single-arm qualitative study explored the diagnostic and treatment planning experience and barriers to treatment participation among children with newly diagnosed CNS tumors and their families. Approved by the Colorado Multiple Institutional Review Board (COMIRB), all participants provided written informed consent; adolescents aged 13-17 provided assent with guardian consent, and those >18 provided their own consent.

Semi-structured interviews captured family experiences and treatment decision-making factors. For patients < 12, only caregivers were interviewed. For ages 13-17, caregivers could choose to include the patient; for those >18, patients could choose to include caregiver(s). This flexible design balanced comprehensive data with minimizing family burden.

Eligible participants included caregivers of children treated at Children’s Hospital Colorado (CHCO) with newly diagnosed or recurrent CNS tumors, as well as patients > 18 meeting the same criteria.

### Data Collection and Interview Process

We identified eligible patients by reviewing pediatric clinic appointments and contacted families by phone to assess interest in Zoom or in-person participation. Families were called up to three times; those not reached were classified as “unreachable” to minimize burden during a stressful period. Interviews continued until thematic saturation was reached at approximately 17 families.

Semi-structured interviews combined guided questions with open-ended discussions. About three months later, follow-up interviews explored the treatment’s impact, how it aligned with expectations, and reflections on the treatment decision.

### Outcome Measures

The primary outcome measure of this study was the identification of barriers to full participation in treatment and clinical trial enrollment, as reported by families during interviews. These barriers could include logistical issues (eg transportation, childcare), financial challenges (eg ancillary expenses), and psychosocial factors (eg emotional stress, social support). The secondary outcome was understanding the experience of families through diagnosis and initial treatment.

### Data Analysis

Interview transcripts were de-identified and analyzed using thematic analysis in NVivo. To ensure inter-rater reliability, two team members (GNH, WD) independently open-coded the first three transcripts, then met to develop a unified codebook. Subsequent interviews were coded by GNH and a second coder (WD or GS) using the codebook. A coding comparison was run by paragraph, and discrepancies were resolved through discussion. The codebook was refined until consensus was reached and all codes had kappa values >0.80. This analysis focuses on themes related to “Experience of Diagnosis,” “Treatment Decision,” “Treatment Decision,” and “Communication.”

## Results

**Figure 1.**
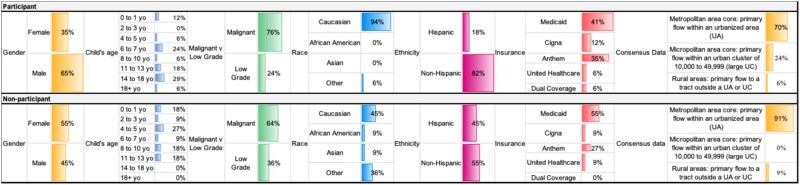
Participant versus non-participant demographics.

A total of 28 patients and families were eligible for this study and invited to participate. Of those, 17 were enrolled and completed interviews. Among the remaining 11, 5 declined participation and 6 were unreachable after 3 attempts. Of the 17 families who participated in the first interview, 13 completed the second interview, while 3 were unreachable after 3 attempts, and 1 patient died before the second interview. Demographic differences between participating and non-participating families are displayed in Figure 1. Notably, the majority of participating families identified as Caucasian, which may reflect participation bias.

**Figure 2.**
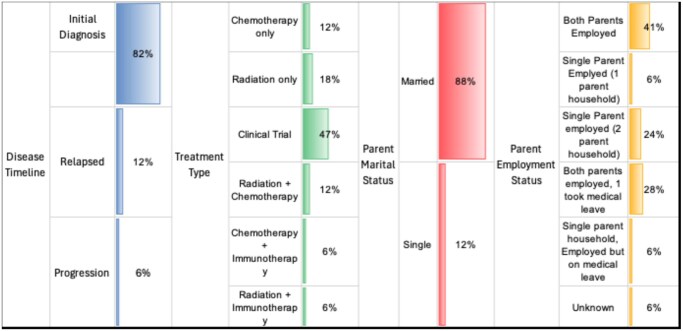
Participant demographics.

Among the 17 families who participated, detailed demographic information—including initial diagnosis vs relapse, treatment type, parental marital status, and parental employment status—is summarized in Figure 2. The average time from diagnosis to study participation was 72 days (median: 69 days; range: 32-184 days). All participating families reported housing stability. The average number of children in each household was 2.65 (median: 2; range: 1-6 children). The race/ethnicity data for the parents were not collected. Additional demographic information, including patients’ ages and details about participation (alone or with a parent), is presented in Table 1. In most cases, the patients’ mother participated in the interviews (*n *= 6). In five interviews, both mother and father (without the patient) participated; in four interviews, the mother and patient participated together. Only the father participated in one interview, and only the patient participated in another. Across all interviews, 28 thematic codes were identified.

**Table 1.** Patient demographics: age and participants

Age group	Participated with parent	Participated alone	Did not participate
13-17	4	0	1
18+	0	1	0
Under 13	0	0	11

Here, we will explore themes related to diagnosis experiences, treatment discussions, treatment decisions, and communication. A comprehensive list of these codes and sub-themes, along with the number of participants who discussed each theme, is shown in Table 2.

**Table 2.** Thematic themes and sub-themes

Themes	Sub-themes	Families (n = 17)	Percentage
**Experience of diagnosis**	Symptoms leading to diagnosis	17	100%
	Delay in diagnosis	5	29%
	Efficiency in diagnosis	8	47%
	Lack of transparency	2	12%
**Treatment discussion**	Confidence in their care team	6	35%
	Difference in opinions among providers	2	12%
	Positives in treatment discussion	6	35%
	Overwhelming or confusing discussion	3	18%
	Families’ recommendations	2	12%
**Treatment decision**	Single option	10	59%
	Confidence in their care team	3	18%
	Support network	4	24%
	Long-term effects	7	41%
	Logistical factors	2	12%
	Financial	1	6%
**Communication**	Positive experiences	7	41%
	Negative experiences	9	53%
	→ Lack of communication and expectations	3 (of 9)	33%
	→ Lack of knowledge or clarity	2 (of 9)	22%
	→ Language barrier	1 (of 9)	11%
	→ Delayed responses	4 (of 9)	44%
	→ Terminology	2 (of 9)	22%
	→ Recommendations	2 (of 9)	22%
	Social challenges	7	41%

### Experience of Diagnosis

All parents discussed their experience of diagnosis, which is further detailed within the subcategories below.

#### Symptoms leading to diagnosis:

All participants named the symptoms that prompted the diagnosis. These symptoms included headaches (7 participants), eye twitching and facial droop (3), accidents with non-healing injuries (2), seizures (1), polyuria (1), vomiting (1), and hematuria (1). The diverse range of symptoms, with varying levels of acuity and severity, led to different experiences in obtaining a diagnosis. One parent reflected on how the ‘vagueness’ of her child’s symptoms delayed seeking care:

*“She came home, and she said that her eye was twitching really fast at school, and it was twitching so fast that it scared her, and then she said that the left side of her tongue was also numb, and, um, she said that the teacher just had her sit down and drink some water ‘cause she thought she was probably tired. And then when it was time for me to pick her up from school, she was completely fine —we didn’t think anything of it.”* (116A, parent)

#### Delay in diagnosis:

Five participants (29.4%) described perceived delays in obtaining a diagnosis. Importantly, these perspectives reflect parental interpretation rather than medically confirmed causality. When symptoms were vague, parents often had to bring their child to multiple appointments without receiving clear answers and advocate for further testing when symptoms persisted. Participants expressed frustration with navigating multiple hurdles, including long wait times for specialist appointments. One parent experienced additional delays due to the holidays, remarking that the waiting was “excruciating.” In one case, the family expressed concern that what they perceived as a delay in diagnosis may have contributed to nerve damage, noting that there “was no sense of urgency, and [that they] had to really advocate” (101A, parent). Several parents also noted prolonged wait times for biopsy results following surgery. Among participating families, 12 resided in urban core areas (RUCA 1), 4 in micropolitan areas (RUCA 4), and 1 in a rural area (RUCA 10). Interestingly, of the families who described delays in diagnosis, the majority lived in urban settings, and no clear pattern emerged based on geographic location.

*“It took about a month to get our diagnosis after that, and then we finally kind of knew that she had cancer. And even that was kind of a struggle to-to get out of [the doctor]. We knew she had cancer because it was taking so long and it was so unorganized. We’re waiting after 10 days, and then two weeks went by and we’re like, ‘Well, I mean, do we call the office ourselves? Should we be diligent and, you know, be active and call?’ So, we would call, and the nurse would say, ‘Oh no, it takes a long time to get in. Like a month.’"* (117A, parent)

#### Efficiency in diagnosis:

In contrast, 8 families (47%) described a swift process from initial symptoms to starting treatment. In these cases, the quick pace provided reassurance and a sense of urgency.

*“I was very relieved when they said, ‘Your surgery is Monday morning,’ She was having surgery within three days. That was very relieving.”* (111A, parent)

While these stories reflect instances when healthcare delivery was efficient, it is important to recognize that the rapid timeline did not always allow families time to process the diagnosis. One parent shared:

*“I would have to say, knowing that it’s a lot to take in, just even the diagnosis, right? That is a huge pill to swallow. Then being told what the care plan is, shortly after, when you don’t even understand how or why, or all the questions you ask yourself. How could this happen? Did I do something? It would have been good to know, like, ‘Here’s the plan of care. We’re going to start with carboplatin and vinblastine, and then we’re going to—you know, it’s going to be hard.’"* (105B, parent)

#### Lack of transparency:

Two families (12%) mentioned experiencing a lack of transparency during the diagnostic process.

*“In my opinion, I thought it was very, very confusing. It almost seemed like they kind of had an idea and knew what it was, but the doctors were more afraid to tell us what it was. But it almost seemed like all the doctors and everybody else knew something that they weren’t telling us, like they were trying to spare us. So, it made it really confusing until we kind of smartened ourselves up and learned what’s going on… if [our child] didn’t tell me she was seeing double vision, we probably would have left this as is for months.”* (117A, parent)

## Treatment Discussion

All seventeen families elaborated on their treatment discussions with the healthcare team. Five themes emerged from these discussions, as outlined below.

### Confidence in their care team:

Six families (35%) expressed confidence and trust in their care team, which guided their treatment decisions.

*“If they didn’t feel comfortable, they didn’t think it was the right thing to do or where it was going to cause her more problems, then we felt that based on their experience and what they do for a living, that we trusted their judgment in that.” (103A, parent)*

*“That’s just what it is, and we have to trust with full-hearted faith that [the doctor] knows what he’s doing. That wasn’t a question. So, we needed his guidance to tell us this is what we’re going to do, and this is why.” (105A, parent)*

### Difference in opinions among healthcare providers:

Two families (12%) experienced differing treatment recommendations between healthcare providers, particularly when these recommendations came from different institutions.

*“On the one hand, you want to have all the options presented to you, but they are so complicated and we’re not experts. There’s a fine line between feeling like we should be the decision makers and then having the doctor say this is our recommendation. The team here has explained a lot of the different options. I think the tricky thing in [my child’s] situation is there are four hospitals involved, and they all disagree.”* (112A, parent)

*“It was really hard for the past couple months trying to take in all of this information that you really need a medical degree to be making these decisions. You’ve got the people with the medical degrees giving us conflicting information, and so, I hope we made the right decision.”* (101B, parent)

### Positives in treatment discussion:

Six families (35%) mentioned positive aspects of treatment discussions, including when doctors took time to answer questions, explain options, and involve the family in decision-making. Families appreciated when the care team used multiple ways to explain the treatment plan, elaborated on the ‘why’ behind their recommendations, and offered second opinions. One family found a timeline roadmap with variations especially helpful for organizing their lives.

*“[The care team] came in, and I remember what was helpful was that they had … the white board, and they wrote down, they were talking to us, but then somebody was there writing notes on the board for us. Here’s what he has. Here’s the type of tumor. Just here’s a couple of websites you can go to, which was nice. Because it’s so many new medical terms that we’ve never heard of before that we wouldn’t even know how to start to spell them. They’re just saying, here you go, here’s what we’re talking about. Then I also liked too that they included us in rounds when they were doing that.”* (110A, parent)

*“I also appreciate how they don’t give you too much information all at once. It’s more digestible, because I don’t know if I'd be able to think about it—worry about this, oh, and three months down the line this is going to happen and this is going to happen. I appreciate that too, and I know my husband does too, that they cut it down into these little bite-sized chunks, which is better to take, because I'm overwhelmed.”* (106A, parent)

### Overwhelming or confusing discussions:

Three families (18%) felt overwhelmed or confused by the information provided during treatment discussions.

*“We have a lot of trust in the doctor, absolutely, but at the same time, understanding what he’s saying is a different thing. We got hit with so much information in such a short period of time we couldn’t even wrap our heads around the diagnosis, let alone his treatment.”* (105B, parent)

*“He came in on a Monday and talked to us for two hours about traditional and conventional chemo versus this clinical trial and all the intricacies. Oh my word, so overwhelmed. When they left, we were just shot, mentally. Still, throughout the week, they would pop in… we spent probably 6 hours discussing and trying to find more information to help us make a decision*.” (101A, parent)

### Families’ recommendations:

Two families offered suggestions for improving the treatment discussion process, advocating for spreading out conversations to allow time for processing. One family also suggested brief check-ins during hospital visits to answer questions or address frustrations.

*“At least let the parents stomach the diagnosis first, talk about the plan, and then give it a couple days to then say, ‘OK, now we need a biopsy. Can we you meet with these people?’ In that way, spread it out, maybe a little bit, just so that the brain and the parents have enough time to comprehend.”* (105B, parent)

## Treatment Decision

Sixteen of the 17 families reflected on the factors that influenced their treatment decisions.

### Single option:

Ten families (59%) mentioned that there was only one clear treatment option, either because it was the standard of care or no other viable alternatives existed.

*“There really wasn’t a choice. It was always just this is what’s happening next because this is the most important piece right now for his health … They laid it all out very well.”* (109A, parent)

*“Radiation was the only option for this type of tumor. It was a little less of a decision, but it was also nice hear that they had just had one picked, and they knew what was best for her. It made the decision easier*.” (102A, parent)

### Confidence in the care team:

Three families (18%) relied on their healthcare team to guide treatment decisions. Among three Hispanic participants, one expressed trust in the care team and relied on providers for treatment decisions. Of 14 White, non-Hispanic participants, five mentioned trust, and two reported depending on provider recommendations.

*“If it was your child, what would you do? Whoever it was, I think, said that they had a six year old, and if it was them, they would do the proton therapy … It was reassuring for someone to take the personal anecdotal aspect of it and say, ‘If it were my child, yes, I would do proton therapy.’”* (111A, parent)

*“I don’t want to tell you what my opinion is, because my opinion doesn’t matter in this world. So yeah, we just wanted [the doctor] to drive [the treatment decision] and tell us.”* (105A, parent)

Six (35%) families explicitly named their confidence in their care team.

*“I have a lot of faith and trust that we were lucky enough to be here, at this hospital, like in their jurisdiction, I guess. Mostly, I thought, like whatever they say is probably the best way to go.”* (107A, parent)

### Support network:

When deciding on treatment, four families (23.5%) considered the importance of staying close to home and having their local support network.

*“Basically, the comforts of home and the support network was a huge factor in our decision to stay here for the surgery … Then, there was that factor of being able to do it here, locally, versus having to get on an airplane, in and out of follow up. That’s a lot, especially in the middle of a pandemic, because this was when we were making the decision, it was early January when Omicron had just spiked. I had to keep both kids out of school. That was an issue all of its own, academically, socially. It was high, high stress.”* (101A, parent)

*“I think it was just knowing that we would be that far away from our family and everybody … That was really the number one was not being so far away from our family. I remember being very, very stressed out about that choice because, of course, it’s down that we could pick the one that had less side effects and possibly better, nicer, but we would have had to go to [out of state] for that. That’s the reason why we did not, because we were so ready to go home and so ready for everybody to be back together.”* (107A, parent)

### Long-term effects:

Seven families (41%) considered long-term effects when making treatment decisions. One parent noted that choosing chemotherapy over surgery was difficult but aimed at ensuring a better future and functional outcome.

*“We ultimately decided on [this doctor] because he seemed to have a more balanced approach of getting out as much tumor as possible but maintaining a neurological function and quality of life.”* (101A, parent)

*“We entered her into [a trial] with the hopes that it was going to prolong her having to do radiation based on her age. Because our fear at that time was just what they had told us with radiation at her age. There could be a lack in cognitive development.”* (103A, parent)

### Logistical factors:

Two families (12%) discussed logistical considerations, such as the number of visits and the travel burden.

*“If we get assigned to the chemo arm of the trial, I think we would bow out of the trial, at that point, because it’s an additional weekly appointment each month, less flexibility … I think, if we had to go a chemotherapy route, we would rather do it conventional versus the clinical trial route just because it would give us more flexibility, one less visit, one less infusion and less time away from school because that’s tricky.”* (101A, parent)

*“Honestly, logistically it was going to probably be a nightmare for us because we were going to have to travel—we live … about four hours [away]. Logistically, it was going to be kind of a nightmare. Because we were going to have to go every other week for like an hour and then come back. We took that into account.”* (103A, parent)

### Financial:

One family mentioned financial concerns, noting the potential burden of travel and the uncertainty around insurance coverage for newer therapies.

*“The proton [therapy] would definitely be more financially prudent. Because you’d have to go to another state [for proton radiation]. You have to find somewhere to stay. If you’re dumping your car, you need to rent a car. There’s a lot more paperwork to go through to figure out—we know Children’s, we know the surrounding campus. It would be something else to learn and more financial burden.”* (109A, parent)

## Communication

All 17 families discussed communication with their care team, sharing both positive and negative experiences. Despite the small sample size, we explored communication experiences by home setting and race/ethnicity. Among 12 urban participants (RUCA 1), 11 discussed communication—64% (*n* = 7) described negative experiences, and 36% (*n* = 4) positive ones. Of four from micropolitan areas (RUCA 4), two reported negative and two positive experiences. The one rural participant (RUCA 10) described a negative experience. Among 14 White, non-Hispanic participants, 12 reflected on communication, evenly split between positive (*n* = 6) and negative (*n* = 6) accounts. Of three Hispanic participants, two described negative experiences, and one reported mixed reflections. These insights also highlight communication challenges beyond the medical team interactions.

### Positive experiences:

Seven families (41%) highlighted positive communication with the care team, appreciating clear, repeated explanations and empathetic providers who set expectations and responded promptly. Several valued having a consistent point of contact; one parent praised weekly texts from clinical navigators. Three families found MyChart, our patient-facing portal that interfaces with EPIC, our medical record, helpful for reaching doctors and getting quick answers.

*“I also feel like they listen to us, and they take time to answer our questions. We feel heard overall l… if we have questions, things that we’re concerned about. They take the time to sit with you and talk to you. They’re empathetic too. They have good bedside manner. We may be the 100th family [this doctor] has seen that day, but for us, it feels like we’re the first because he gives that much attention and care to our son and to us as a family. That means a lot.”* (106A, parent)

*“The positives were the way the doctors explained it like why that treatment was the best. They gave you the side effects that could happen and the side effects that were less likely to happen. Just gave you a lot of information. They were very informational about it.”* (102A, parent)

### Negative experiences:

Nine families (53%) identified at least one negative aspect within communication.

### Lack of Communication and Expectations

Three families wanted clearer communication about expectations and next steps. One cited unclear guidance on rehab stay and recovery; another felt unprepared for the length and side effects of treatment.

*“It’s such an emotional roller coaster. I feel like we have been just blindsided time and time again. Part of it, I think, would be due to lack of communication from the [doctor’s] team. Maybe part of it is unrealistic expectations on my part. First of all, we are really hoping that we could get the entire tumor. In my mind, I was thinking, if not 100% then 80%? To learn it was 50 was a blow. Then, we were told to expect three-to-five day recovery in the hospital … When we got checked into rehab, they told us that the average stay was four to six weeks. That was another just hit with a ton of bricks, wasn’t expecting that. Had they just communicated, I would have been better mentally prepared.”* (101A, parent)

Another family felt frustrated by the lack of transparency regarding side effects, frequency of visits, and life after treatment, making it difficult to plan family travel, school, and the future.

*“I don’t think there’s a landscape of endless opportunities here. I think we generally know what we’re dealing with, I think. If there is some uncertainty, they need to say, ‘Hey, we’re not sure, but best guess is something’s going to happen between two and four weeks, or we do this every odd Friday.’ They know more than they’re telling me. I don’t know why no one is telling me when I ask questions and it’s completely just saying nothing.”* (114A, parent)

A third family noted that it took a month for the care team to explicitly say “cancer,” by which time the parents had already inferred it from the clinical notes.

*“Not knowing what the heck was going on, so that was one of the most frustrating parts. And, you know, as a parent you’re sitting there for all that time, we’re just worrying, worrying, worrying about what to expect, you know?”* (116A, parent)

### Lack of Knowledge or Clarity

Two families discussed challenges due to their limited knowledge.

*“I wish I would have known more, so I could ask more questions. Knowing more, like if the infusions didn’t work, what our next steps would be. What are the long-term side effects? Just more detailed information. We got hit with so much information in such a short period of time. We couldn’t even wrap our heads around the diagnosis, let alone his treatment.”*(105B, parent)

Another family wanted clarity on whether doctors believed the chemotherapy was working, noting that parents see only side effects and need reassurance about treatment effectiveness.

### Language Barrier

Communication was also challenging for non-English-speaking families. One bilingual patient shared that, because the translator took too long to arrive for appointments, she often had to explain medical details to her mother.

*“It has been difficult for both of us because sometimes I don’t know the word in Spanish or in English, and then it’s difficult for me to translate for my mom. We’ve made it work.”* (104A, patient)

### Delayed Responses

Four families reported delays in communication, citing challenges like phone tag and slow portal responses. One parent noted that while responses were prompt during active treatment, contact became harder once they left the hospital. The parent stated, “At times it felt like out of sight, out of mind.” (113A). Others noted receiving responses from different nurses in the same message chain, without any one person providing an answer. Two families expressed frustration with the volume of information sent through the portal, including messages, appointment notifications, and resources. This contributed to what they called “MyChart fatigue.”

### Terminology

Two families found medical terminology confusing. One felt misled by interchangeable terms like “mass,” “tumor,” and “lesion.” Another wished they had pushed for further care sooner but were falsely reassured by terms like “enlargement,” “lump,” or “swelling.”

### Recommendations

Two families offered suggestions to improve communication. One recommended assigning a point of contact to relay questions and ensure timely responses. Another emphasized personalized discussions about next steps, including school and work, and suggested that care teams consider each family’s circumstances when offering guidance. Families also wanted more clarity on appointment and scan schedules, noting that a draft calendar would aid planning and restore a sense of normalcy.

#### Social challenges

Seven families struggled to discuss the illness outside the healthcare setting. Three parents found it challenging to talk to their child about the diagnosis; one suggested that doctors offer a high-level explanation and be available for follow-up questions. Another parent noted discomfort discussing complications, such as possible paralysis, diaper use, and fertility issues like sperm banking. Two parents also struggled to explain the diagnosis to friends and family.

*“A lot of people don’t understand. Only certain people I feel comfortable talking to. And then there’s certain people I don’t. Sometimes, I just don’t want to talk at all. That was the hardest thing in the beginning, I just didn’t want to talk to anybody. I just really wanted to process for myself, and a lot of people didn’t understand that. Some people still have hard feelings over that, but I feel like they’re not in my shoes. They can’t understand. Sometimes, you just need to take time for yourself to process it yourself.”* (102A, parent)

When another family explained their child was diagnosed with a brain tumor, they were met with pity and sadness in others’ eyes, which made it more difficult for them when they were hoping for strength.

Two additional families struggled with explaining to younger siblings in an age-appropriate way. Parents felt uncertain about how much information to share with parents of their child’s friends, wanting to respect their child’s privacy but also address the inevitable questions. Both participants wished for more help from the care team on language that could possibly help other kids understand.

## Discussion

Our study highlights the complexities families encounter during the diagnosis, decision-making, and early treatment of pediatric central nervous system tumors. While reinforcing established themes in the literature, we also identify areas for further exploration and improvement. Consistent with previous research, families described the emotional toll of navigating a diagnosis.^7^^,^^8^ However, our findings offer deeper insights into the nuances of communication, treatment discussion, and decision-making. While limited research addresses communication and care quality in pediatric neuro-oncology, related studies in both pediatric and adult settings have examined health-related quality of life and caregiver burden. Caregivers of children with brain tumors often report significant stress and uncertainty, particularly around symptom management, prognosis communication, and care coordination.^9^ However, most research emphasizes clinical outcomes or symptom-specific quality of life rather than broader perceptions of care at key timepoints such as diagnosis.^10^ Our study builds on this work by uniquely capturing caregiver perspectives on communication, support systems, and informational needs during early treatment. By focusing on diagnosis and initial care, we shed light on a critical but understudied period that shapes family experience and trust in the care team. This work identifies actionable communication and system gaps not addressed by traditional quality of life frameworks.

The diagnostic phase was fraught with emotional distress, as families grappled with shock, uncertainty, and a search for answers. Many experienced delays due to misinterpreted symptoms or inadequate initial evaluations, heightening frustration and anxiety. In contrast, families of patients with “red flag” symptoms, such as seizures, often reported a more efficient diagnostic process, highlighting variability in timeliness and care experiences. The way a diagnosis was framed, the timing of information delivery, and the sensitivity of the initial communication profoundly influenced families’ trust in the medical team and their readiness for informed decision-making. These findings emphasize the need for provider training in delivering difficult news while both transparency and emotional support. Tailoring diagnosis delivery to each family’s needs—while ensuring immediate access to coping resources—could significantly enhance their experience and engagement in care.

When reflecting on treatment discussions and decisions, families described both positive and negative experiences. They valued providers who explained treatment options thoroughly and empathetically, using clear language, multiple explanations, and well-defined expectations. This aligns with existing literature on the importance of shared decision-making and transparent communication in building trust.^11^^,^^12^

A key insight from our findings is the need for tailored communication strategies that account for families’ varying levels of health literacy and emotional readiness. Many expressed a desire for clearer expectations regarding treatment timelines, side effects, and logistics, suggesting that a more structured approach could enhance their preparedness and engagement. Conversely, gaps in clarity often resulted in negative experiences. Another challenge was conflicting recommendations among healthcare providers, which created uncertainty and weakened families’ confidence in decision-making. This underscores the importance of improved coordination and consensus-building among specialists to present unified guidance. Additionally, discussions overloaded with medical jargon or excessive information left families struggling to process key details, limiting their ability to engage meaningfully. To improve treatment discussions, families recommended spacing conversations over multiple sessions with brief check-ins to allow time for processing and questions. This iterative approach could ease cognitive and emotional burdens while keeping families informed and engaged throughout the care journey. Implementing these strategies may significantly enhance communication quality and strengthen the family-care team relationship.

The theme of “Treatment Decision” revealed the diverse ways families navigate decision-making, shaped by their unique priorities and circumstances. Some families faced a single treatment option, simplifying the process, while others relied on their care teams’ expertise, placing full trust in their doctors. Other key treatment considerations included proximity to home, support networks, long-term side effects, and logistical challenges such as treatment frequency and financial burdens. These findings underscore the multifaceted nature of treatment decision-making and the importance of providing clear, personalized guidance that aligns with each family’s specific priorities and concerns.

The final theme, communication, underscored its vital role in building trust and partnership. Families valued providers who demonstrated empathy, promptly addressed concerns, and fostered an unhurried environment for discussion. Communication was most effective when delivered through multiple formats—verbal explanations, written summaries, and visual aids—allowing families to process and revisit information in ways that suited their needs. In contrast, delays and lack of transparency with diagnosis or treatment course exacerbated stress and hindered decision-making. A notable finding was the need for a consistent point of contact within the care team, a role that could mitigate communication challenges such as slow responses and fragmented messaging. This reinforces and expands on existing recommendations for family-centered care models and streamlined communication channels to enhance continuity and reduce the burden on families.^13^

Our findings highlight several actionable steps to enhance the family experience. First, providers should prioritize proactive communication by anticipating concerns and clearly outlining potential outcomes, treatment side effects, and next steps in a transparent, timely manner. Secondly, although care coordinators are often part of the neuro-oncology multidisciplinary team, newly diagnosed families in the inpatient setting may be unaware of these resources. Our findings suggest that introducing care coordinators early during inpatient hospitalization could enhance communication and provide families with a consistent point of contact throughout treatment. Additionally, communication barriers may prevent families from learning about clinical trial opportunities, potentially limiting their child’s treatment options. Introducing clinical trial discussions earlier in the process and ensuring families fully understand participation implications could improve enrollment and access to innovative care.

Further research is essential to generalize these findings and translate them into practice. Our study lays the groundwork for quantitative investigations into family satisfaction with communication and decision-making in pediatric neuro-oncology. Surveys could assess families’ experiences with communication, access to treatment information, and understanding of treatment options. Additionally, comparing trial enrollment rates between families who receive early, consistent communication about these opportunities and those who do not could reveal the impact of communication strategies on participation and identify barriers to clinical trial enrollment. Future interventions should focus on improving access to treatments, reducing disparities, and enhancing the family experience throughout diagnosis and care. Additionally, we could not account for provider factors (eg training, experience) that may have influenced communication and planning. Finally, this study was conducted at a single institution with a participant population that was predominantly white, which may limit the generalizability of our findings and underrepresent the experiences of families from marginalized or underserved communities. Future research should prioritize strategies to better engage underrepresented families to ensure more equitable representation.

## Data Availability

The datasets generated during and/or analyzed during the current study are available from the corresponding author on reasonable request.
